# Vomeronasal and Olfactory Structures in Bats Revealed by DiceCT Clarify Genetic Evidence of Function

**DOI:** 10.3389/fnana.2018.00032

**Published:** 2018-05-08

**Authors:** Laurel R. Yohe, Simone Hoffmann, Abigail Curtis

**Affiliations:** ^1^Department of Ecology & Evolution, Stony Brook University, Stony Brook, NY, United States; ^2^Department of Anatomy, New York Institute of Technology, College of Osteopathic Medicine, Old Westbury, NY, United States; ^3^Department of Biology, University of Washington, Seattle, WA, United States

**Keywords:** vestigial, vomeronasal organ, main olfactory system, chemosensation, diceCT

## Abstract

The degree to which molecular and morphological loss of function occurs synchronously during the vestigialization of traits is not well understood. The mammalian vomeronasal system, a sense critical for mediating many social and reproductive behaviors, is highly conserved across mammals. New World Leaf-nosed bats (Phyllostomidae) are under strong selection to maintain a functional vomeronasal system such that most phyllostomids possess a distinct vomeronasal organ and an intact TRPC2, a gene encoding a protein primarily involved in vomeronasal sensory neuron signal transduction. Recent genetic evidence, however, shows that TRPC2 is a pseudogene in some Caribbean nectarivorous phyllostomids. The loss-of-function mutations suggest the sensory neural tissue of the vomeronasal organ is absent in these species despite strong selection on this gene in its mainland relatives, but the anatomy was unknown in most Caribbean nectarivorous phyllostomids until this study. We used diffusible iodine-based contrast-enhanced computed tomography (diceCT) to test whether the vomeronasal and main olfactory anatomy of several phyllostomid species matched genetic evidence of function, providing insight into whether loss of a structure is linked to pseudogenization of a molecular component of the system. The vomeronasal organ is indeed rudimentary or absent in species with a disrupted TRPC2 gene. Caribbean nectar-feeders also exhibit derived olfactory turbinal morphology and a large olfactory recess that differs from closely related bats that have an intact vomeronasal organ, which may hint that the main olfactory system may compensate for loss. We emphasize non-invasive diceCT is capable of detecting the vomeronasal organ, providing a feasible approach for quantifying mammalian chemosensory anatomy across species.

## Introduction

Vestigial structures are commonly observed across animals, but the molecular and morphological underpinnings of the process of trait loss through time is not well understood (Fong et al., 1995; Lahti et al., 2009). The loss of sensory systems is frequently discussed in the context of vestigialization, with the loss of vision in cave-dwelling fish being a common example (Jeffery et al., 2003). Selection may be relaxed on senses that no longer contribute to fitness, and sensory organs may become vestigial (Fong et al., 1995). Loss is often attributed to the irrelevance of the sensory system to the functional ecology of the species (Lahti et al., 2009). However, loss of a sensory system may not translate into complete loss of function. Bottlenose dolphins (*Tursiops truncatus*), for example, have degraded or absent taste buds (Kastelein and Dubbeldam, 1990), and many of their taste receptor genes contain loss-of-function mutations (Jiang et al., 2012). Yet, several behavioral studies suggest this species can still decipher an abundance of flavor profiles (reviewed in Kremers et al., 2016a,b). Male mandrills (*Mandrillus sphinx*) flaunt secretions from their sternal gland during dominance displays, though the organ that supposedly processes these social chemical cues is vestigial (Setchell et al., 2010). How sensory structures are lost without complete loss of the sense, and possible mechanisms of sensory compensation remain largely unknown. Comparative analyses of the molecular and morphological components of sensory systems can clarify these mechanisms of relaxed selection, as they provide insight into when shifts in selection may have occurred. In this study, we integrated molecular and morphological evidence for loss of function, connecting the process of pseudogenization of genes and vestigialization of morphology.

The nasal chemosensory system is a primary sensory modality for most mammal species and it is critical to mediating many behaviors essential to fitness, including finding food, identifying conspecifics, avoiding noxious chemicals, detecting predators, and caring for offspring (Ache and Young, 2005; Fortes-Marco et al., 2013; Liberles, 2014). These behaviors are responses to environmental chemical signals processed by two major chemosensory organs in the nose. First, the olfactory epithelium lines part of the nasal cavity and expresses olfactory receptors that bind to volatile chemical ligands from the environment (Hayden and Teeling, 2014). Second, the vomeronasal organ is a tube-shaped structure located in the anteroventral region of the nose lined with epithelial tissue that is thought to primarily detect pheromonal chemical cues (Liberles, 2014). These pheromonal cues are important for social communication and reproduction and are typically non-volatile (Eisenberg and Kleiman, 1972; Liberles, 2014). Recent evidence has increasingly suggested the functions of the two nasal chemosensory systems are not as dichotomized as previously thought, and a more complex and interdependent relationship between the two exists from an anatomical, molecular, developmental, and neurobiological level (Keller et al., 2009; Baum, 2012; Keller and Lévy, 2012; Suárez et al., 2012; Huilgol et al., 2013; Hohenbrink et al., 2014; Omura and Mombaerts, 2014; Baum and Cherry, 2015). There is extensive variation in morphology and genetic diversity in mammalian chemosensation (Hillenius, 1992; Hayden et al., 2010), but the sensory systems are well-conserved in terms of function given the essential role of the main olfactory and vomeronasal system in mediating behaviors relevant to reproduction and survival (Grus et al., 2005; Grus and Zhang, 2009; Ibarra-Soria et al., 2014).

**FIGURE 1:**
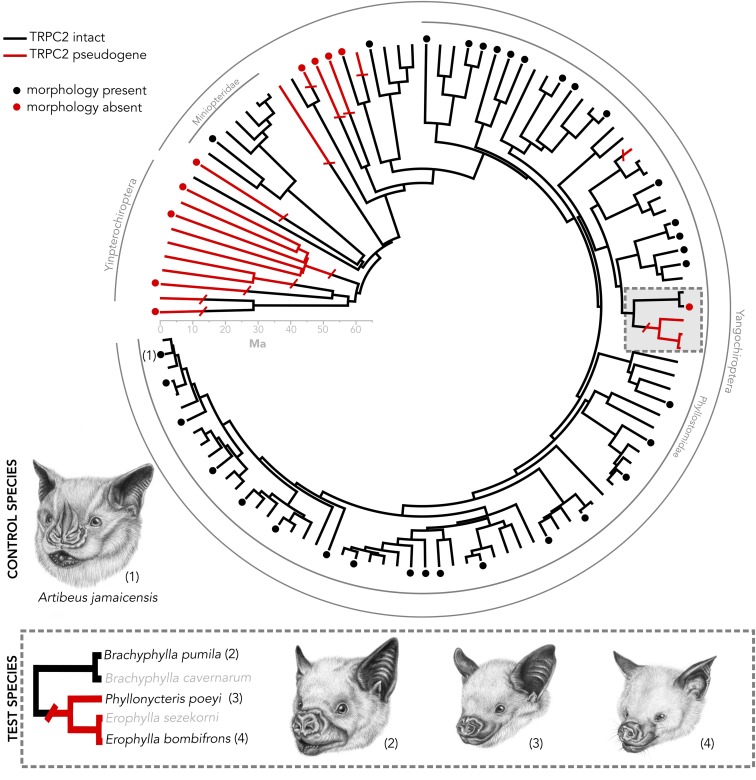
Species tree of bats in which the *Trpc2* gene has been sequenced from a previous analysis (Yohe et al., 2017). Numbered taxa are the four species used in this study. Caribbean nectar-feeding bats serve as a model in which we tested whether vomeronasal morphology matches *Trpc2* function. *Artibeus jamaicensis* serves as a control species, as it has known intact vomeronasal morphology and intact *Trpc2*. Dots indicate taxa in which vomeronasal morphology is known. The presence of vomeronasal morphology may indicate either the presence of a vomeronasal organ, accessory olfactory bulb, or both. All illustrations were done by Elissa Johnson.

Despite its relevance to fitness, most bats have independently lost function of the vomeronasal system, with evidence from both morphological and molecular data (**Figure 1**) (Bhatnagar and Meisami, 1998; Meisami and Bhatnagar, 1998; Yohe et al., 2017). Numerous histological studies have shown that dozens of species have rudimentary or absent vomeronasal morphology (Cooper and Bhatnagar, 1976; Frahm and Bhatnagar, 1980; Wible and Bhatnagar, 1996; Bhatnagar and Meisami, 1998; Meisami and Bhatnagar, 1998). Previous work has demonstrated from sequencing the second exon of the gene Transient receptor potential cation channel 2 (TRPC2), a gene that encodes an ion channel that transduces the pheromone signal in vomeronasal neurons, that it is pseudogenized in many species with a vestigial vomeronasal organ (Zhao et al., 2011; Yohe et al., 2017), but whether this molecular loss of function is coupled with complete anatomical vestigialization is not well understood. Furthermore, though many bats have lost function of their vomeronasal system, they still retain social behaviors that are mediated by chemical cues, suggesting a role of the main olfactory system. For example, *Erophylla* performs wing-flapping dances and sprays garlic-scented secretions from the supraorbital glands when courting a female (Murray and Fleming, 2008). The mechanisms that enable pheromone communication in the absence of the vomeronasal organ are a new frontier in neuroscience. Recent research of vomeronasal and TRPC2 knockouts in rodents have unveiled ways in which some of these behaviors may still be mediated by alternative ion channels or the main olfactory system (Pankevich, 2004; Chamero et al., 2012; López et al., 2014; Omura and Mombaerts, 2014; Yu, 2015). However, looking beyond the rodent model, the natural independent vestigialization of the vomeronasal organ coupled with observed intact behavior, widespread throughout the bat phylogeny, provides the opportunity to test alternative mechanisms of sensory loss, as the actual signals seem to still be processed by the organisms and not truly “lost.”

We present a system of bats that sets up framework for exploring mechanisms surrounding the vestigialization and pseudogenization of a sensory system. While most bat families do not possess an intact vomeronasal organ, there are three curious exceptions, including New World Leaf-nosed bats (Phyllostomidae), the closely related genus *Pteronotus* (Mormoopidae), and Long-fingered bats (Miniopteridae) (**Figure 1**). A recent study on the molecular evolution of TRPC2 showed that these two families and *Pteronotus* have been under invariantly strong purifying selection to maintain a functional ion channel since the common ancestor of all bats (Yohe et al., 2017). However, a recent shared ancestral loss of TRPC2 was observed in Caribbean nectar-feeding phyllostomids (**Figure 1**) that potentially suggests the vomeronasal system is non-functional, despite the notion that its mainland relatives are under strong selection to keep the gene intact. This clade, known as Brachyphyllini (*Brachyphylla, Erophylla*, and *Phyllonycteris*), is a monophyletic group of nectarivorous bats endemic to the West Indies that colonized the western islands and spread east, some even into the Lesser Antilles (Carstens et al., 2004; Dávalos, 2004; Murray, 2008). The morphology of the vomeronasal system is unknown in *Erophylla* and *Phyllonycteris*, though these bats are known to have social behaviors mediated by chemical cues suggesting that a mechanism to relay these social chemical cues still exists in these bats (Murray and Fleming, 2008), whether it be an intact vomeronasal organ or compensation by the main olfactory system. Interestingly, the sister taxon *Brachyphylla* has an intact TRPC2 but the vomeronasal organ is described as rudimentary (Bhatnagar and Meisami, 1998). Other molecular components of the system, such as the vomeronasal receptors, are of unknown function for brachyphyllines. This asynchronous pattern of loss sets up a compelling system to connect pseudogenization with vestigialization, as well as to look for sensory compensation by the main olfactory system.

We integrate recent genetic evidence of function with a new non-destructive sampling method of morphology called diffusible iodine-based contrast-enhanced computed tomography (diceCT) (Gignac and Kley, 2014; Gignac et al., 2016). DiceCT makes use of the contrast-enhancing staining agent Lugol’s iodine that increases the radiodensities of soft tissues for micro-computed tomography (μCT) scanning, thus enabling quantification of soft tissue anatomy without destruction of the specimen. While diceCT certainly cannot replace the resolution and detail of serial histological sectioning, it provides several advantages in the context of our study. DiceCT is a reversible staining technique that is minimally destructive to fixed specimens, allowing the possibility to explore soft tissue anatomy of rare species. It offers the benefit of reconstructing morphology of obscure soft tissue structures in three dimensions. DiceCT is also time-efficient and opens up the possibility of large-scale comparative analyses and the ability to analyze several samples in a series. Using diceCT, we identified target nasal chemosensory structures, including the epithelium lining the nasal cavity and the vomeronasal organ. This is the first evidence of quantifying these structures using diceCT. We sampled all three genera within Brachyphyllini (**Figure 1**) that show genetic evidence of loss of function and one distantly related frugivorous phyllostomid with known vomeronasal function from both genetics and morphology (*Artibeus jamaicensis*; Bhatnagar and Kallen, 1974; Yohe et al., 2017). If Caribbean nectar-feeding bats have lost function of TRPC2 and also lack the vomeronasal organ, then these bats have lost complete function of their vomeronasal system and must relay social chemical cues via another sensory system. Alternatively, if Caribbean nectar-feeding bats have a disrupted TRPC2 but have intact vomeronasal morphology, then these bats must be using an alternative signal transducing mechanism in the vomeronasal organ. In light of the presence of chemically-mediated social behaviors in this group, we also describe and quantify tissues of the main olfactory system, and propose several hypotheses as to how the main olfactory system may compensate for vomeronasal loss.

## Materials and Methods

### Samples

This study targeted species that reflect the functional diversity of both vomeronasal form and TRPC2 variation. *Artibeus jamaicensis*, a species used as a positive control, is a frugivorous phyllostomid that has a well-developed vomeronasal organ known from histological evidence and an intact and well-conserved TRPC2 sequence (**Figure 1**; Bhatnagar and Kallen, 1974; Yohe et al., 2017). Identification of vomeronasal structures based on diceCT data in this specimen would indicate the method is sufficient for detecting vomeronasal structures. We compared our diceCT scans with previously published histological sections of the nasal cavity of *A. jamaicensis* (Eiting et al., 2014). Within the nectarivorous phyllostomid clade Brachyphyllini, we sampled the following taxa: *Brachyphylla pumila* that has an intact TRPC2 and the anatomy is unknown but whose sister species (*B. cavernarum*) has a rudimentary vomeronasal organ (Bhatnagar and Meisami, 1998); *Erophylla bombifrons* and *Phyllonycteris poeyi*, two species that have a non-functional TRPC2 but the anatomy is unknown. One specimen per aforementioned species was collected in the Dominican Republic during a February 2015 expedition and scarified using isoflurane following the approved animal care protocols at Stony Brook University (IACUC 554555). Each specimen was fixed in 4% buffered formalin until iodine-staining.

### Iodine-Staining and μCT-Scanning

After fixation, the four specimens (one per species) were decapitated and placed in 11.25% iodine-potassium iodide (I_2_KI) solution within 1 week of returning from the field expedition. Specimens remained in the same solution for ∼10–15 weeks. Prior to μCT scanning, the stained specimens were washed in distilled water to remove excess I_2_KI solution and gently patted dry with a paper towel. Excess liquid was gently pipetted from the nasal chamber. Individual specimens were wrapped in ziplock bags to prevent desiccation during scanning. The nasal region of all specimens was scanned at the American Museum of Natural History using a GE V| tome| x μCT scanner using the 240 kV micro-focus high energy x-ray tube. Following Gignac and Kley (2014), the x-ray tube was fitted with a 0.1 mm copper filter. Isometric voxel sizes (mm), voltage (kV), and current (μA), depended on the dimensions and radio-opacity of each specimen, and these values are provided in **Table 1**. The TIFF stacks were aligned in VG Studio^1^ so that they fell along the sagittal, transverse, and frontal planes for each specimen, with the palate and nasal septum defining the frontal and sagittal planes, respectively. Aligning scans to these standard anatomical planes eased identification of homologous structures and allowed for more consistent segmentation of the vomeronasal organ and homologous turbinal elements. The aligned μCT slices were exported as 16-bit TIFF files.

**Table 1:** μCT scanning parameters used for the rostral scans of each specimen in the analysis.

Species	Voxel Size (mm)	Voltage (kV)	Current (μA)	Filter
*Artibeus jamaicensis*	0.0196	150	120	0.1 mm Cu
*Erophylla bombifrons*	0.0149	110	130	0.1 mm Cu
*Phyllonycteris poeyi*	0.0194	150	120	0.1 mm Cu
*Brachyphylla pumila*	0.0207	130	150	0.1 mm Cu

*Full documentation for each scan is available on MorphoSource.*

### Anatomical Reconstructions

Aligned TIFF stacks were imported into Amira 6.0 (FEI Thermo Fisher Scientific) for segmentation and volumetric rendering of the nasal cavities. The vomeronasal organ and olfactory turbinals (including the turbinal bones and associated mucosae) were segmented using the threshold function and manual segmentation tool. Labels were smoothed in Amira using the “Smooth labels in 3D” function. Three-dimensional surface models were generated for all specimens using the “Generate Surface” module in Amira. We follow the terminology used by previous studies naming turbinals after the bone with which they articulate (e.g., frontoturbinal, ethmoturbinal) (Maier, 1993; Smith and Rossie, 2006, 2008). Turbinals that arise from the medial wall of the orbitonasal lamina and are concealed from medial view by ethmoturbinals are referred to as interturbinals. Only the frontoturbinal, ethmoturbinals, and interturbinals were segmented in this study, as they hold a majority of the olfactory tissue in mammals (Van Valkenburgh et al., 2014). Turbinals were color-coded to reflect hypothesized homologies.

### Measurements

Volumetric and surface measurements were calculated in Amira using the “Surface Area Volume” module. It should be noted that the surface area measurements of turbinals from volumetric models are estimated differently from surface measurements of turbinals from histological studies and are not directly comparable. Additionally, olfactory turbinal surface area is not an accurate proxy for olfactory epithelial surface area due to olfactory turbinals being only partially covered in olfactory epithelium and epithelial distribution varies among taxa. However, other studies suggest that olfactory turbinal surface area is an acceptable general estimate of olfactory epithelial coverage (Van Valkenburgh et al., 2011). Linear measurements of the skull, transverse lamina, nasal cavity, and vomeronasal organ were taken in Amira using the 3D measurement tool (see Supplementary Figure S1 for details on transverse lamina and nasal cavity length measurements).

## Results

### Vomeronasal Organ

**FIGURE 2:**
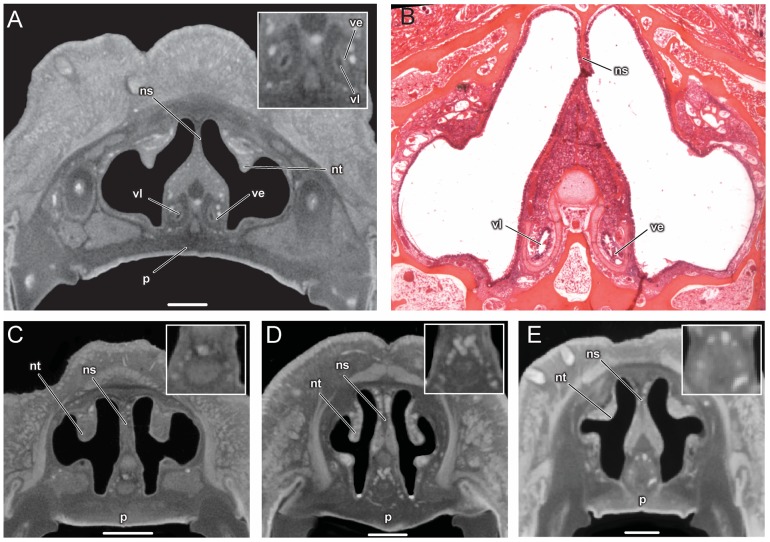
Comparison of coronal sections from the anterior part of the nasal cavity based on diceCT scans **(A)** and corresponding histological section for **(B)** of *Artibeus jamaicensis*, and corresponding diceCT slices for **(C)** *Erophylla bombifrons*, **(D)** *Phyllonycteris poeyi*, and **(E)** *Brachyphylla pumila*. Sections were taken just posterior to the opening of the nasopalatine duct. Insets show magnified image of the region of the vomeronasal organ. Scale 1 mm. The vomeronasal organ is clearly visible in the diceCT scan and histological section of *Artibeus* **(A,B)**, but not in *Phyllonycteris*, *Erophylla*, and *Brachyphylla* **(C–E)**. ns, nasal septum; nt, nasal turbinal; p, palate; ve, vomeronasal epithelium; vl, vomeronasal lumen.

**FIGURE 3:**
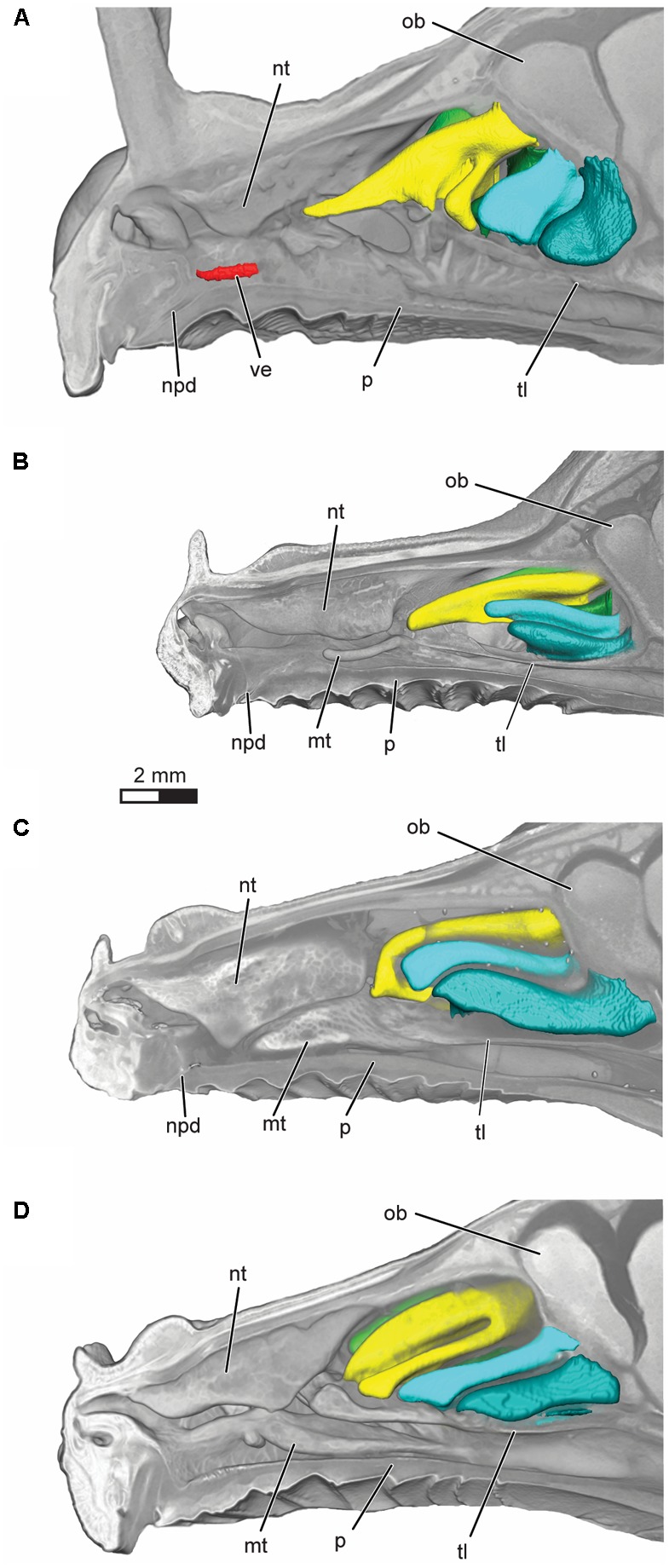
Volume rendering of the nasal cavity in mid-sagittal cut-away view of right turbinals and vomeronasal organ in **(A)** *Artibeus jamaicensis*, **(B)** *Erophylla bombifrons*, **(C)** *Phyllonycteris poeyi*, and **(D)** *Brachyphylla pumila*. Turbinals are colored to reflect homology; frontoturbinal (light green), interturbinals (dark green), ethmoturbinal I (yellow), ethmoturbinal II (light blue), and ethmoturbinal III (teal). Vomeronasal organ is colored in red. Position of the transverse lamina label indicates first slice in which the lamina is complete, demarcating the beginning of the olfactory recess. ob, olfactory bulb; mt, maxilloturbinal; npd, nasopalatine duct; nt, nasoturbinal; p, palate; tl, transverse lamina; ve, vomeronasal epithelium.

The vomeronasal organ is clearly visible in the diceCT scans of *Artibeus jamaicensis* (**Figures 2A**, **3A**). Its overall appearance matches histological observations described in previous studies (**Figure 2B**; Bhatnagar and Kallen, 1975; Cooper and Bhatnagar, 1976; Bhatnagar and Meisami, 1998; Eiting et al., 2014). The vomeronasal epithelium and lumen can be distinguished in diceCT scans. Based on the surface reconstruction, the vomeronasal organ of *A. jamaicensis* is 1.7 mm long (anteroposteriorly) and 0.3 mm tall (dorsoventrally). The volume of the epithelium is 0.07 mm^3^ and the surface area of the epithelium is 2.1 mm^2^. The greatest length is similar to the length given by Cooper and Bhatnagar (1976) of 1.6 mm. This demonstrates that the vomeronasal organ can be accurately reconstructed from diceCT scans without any substantial shrinkage. The vomeronasal organ was not detectable in the diceCT scans of *B. pumila, E. bombifrons*, and *P. poeyi* (**Figures 2C–E**, **3B–D**, and Supplementary Figures S3–S5), indicating that the organ is, at best, rudimentary in Brachyphyllini. This is congruent with Bhatnagar and Meisami’s (1998:4I) identification of a possible rudimentary vomeronasal organ dorsal to the paraseptal cartilages in *B. cavernarum*.

### Identifying Olfactory Epithelia With DiceCT

**FIGURE 4:**
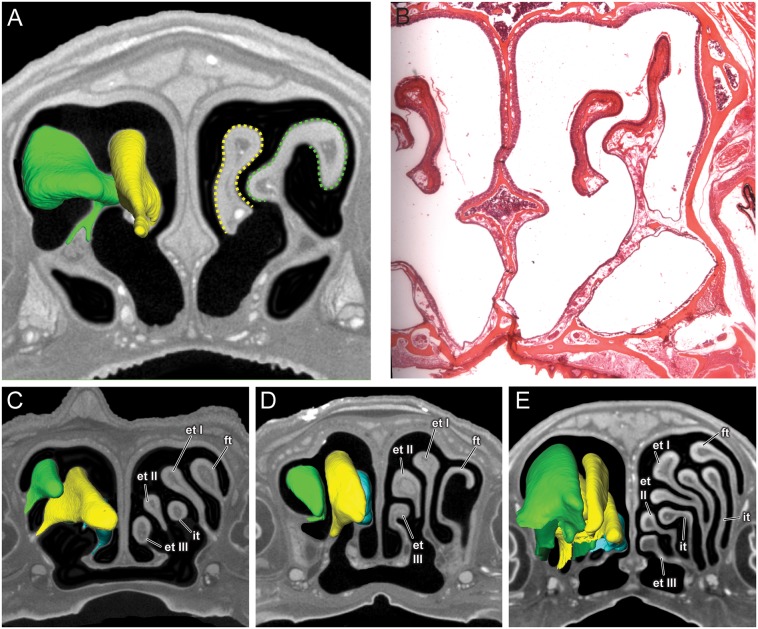
Coronal sections of the posterior region of the nasal cavity comparing diceCT scans **(A)** and corresponding histological section **(B)** for *Artibeus jamaicensis*, and corresponding diceCT slices for **(C)** *Erophylla bombifrons*, **(D)** *Phyllonycteris poeyi*, and **(E)** *Brachyphylla pumila*. Three-dimensional renderings of right olfactory turbinals are colored to reflect homology; frontoturbinal (light green), interturbinals (dark green), ethmoturbinal I (yellow), ethmoturbinal II (light blue), and ethmoturbinal III (teal). Olfactory epithelium lining the turbinals indicated by dotted lines in *Artibeus* (A). et I, ethmoturbinal I; et II, ethmoturbinal II; et III, ethmoturbinal III; ft, frontoturbinal; it I, interturbinal I; it II, interturbinal II.

In histological sections, the olfactory epithelium is readily identified based on a combination of features, including presence of Bowman’s gland, non-motile cilia, and clear separation into three cell layers (Farbman, 2000). None of these traits are detectable in the diceCT scans of these four bats. However, in *A. jamaicensis*, the changes in thickness of the epithelium covering the turbinals seem to reasonably track the occurrence of olfactory epithelium known from histology (**Figures 4A,B**; see Figure 7 in Kämpfer and Schmidt, 1977). Identifying the boundary between olfactory epithelium and respiratory epithelium along the nasal septum is, however, much more uncertain. Respiratory mucosa can be readily identified in the anterior part of the nasal cavity due to the presence of numerous large mucosal glands, but the transition between olfactory and respiratory epithelia posteriorly is not immediately apparent. It should be noted that only histological sections of *A. jamaicensis* were available to us. It is possible that olfactory epithelium might be easier to identify along the nasal septum in the μCT scans of brachyphyllines, but without comparison to histological sections we cannot distinguish the olfactory from the respiratory epithelia with any degree of certainty. As such, we are taking a more conservative approach and only compare the size and shape of the frontoturbinals, ethmoturbinals, and interturbinals rather than estimating olfactory epithelial area.

### Turbinals

All four bats possess a frontoturbinal, three ethmoturbinals (ethmoturbinal I–III), and one interturbinal (interturbinal II) between ethmoturbinal I and ethmoturbinal II on each side (**Figures 3**, **4**; see also Supplementary Figures S2–S5). *B. pumila* has an additional interturbinal, between the frontoturbinal and ethmoturbinal I (**Figure 4**), here referred to as interturbinal I. Identification of these turbinals differs from that of Bhatnagar and Kallen (1974, 1975) and Kämpfer and Schmidt (1977) and is listed in **Table 2**. All linear, surface area, and volume measurements of turbinals are given in **Table 3** and linear measurements of the nasal cavity are given in **Table 4**. The morphology of the turbinals of Brachyphyllini differs from *A. jamaicensis* in several aspects. First, the frontoturbinals and interturbinals form single folds in Brachyphyllini, whereas they are double-scrolled and t-shaped in *A. jamaicensis*. Second, ethmoturbinal II and III are anteroposteriorly elongate and extend further anteriorly in Brachyphyllini (28–32% and 26–37%, respectively of nasal cavity length; **Table 4**), whereas they are stout and anteroposteriorly shorter in *A. jamaicensis* (19 and 20%, respectively, of nasal cavity length; **Table 4**). Third, the transverse lamina extends farther anteriorly in Brachyphyllini resulting in a larger olfactory recess than in *A. jamaicensis* (**Figure 3** and **Table 4**). The ratio of the transverse lamina to nasal cavity length in *P. poeyi* (0.32) and *B. pumila* (0.29) is almost twice that of *A. jamaicensis* (0.17). This change in olfactory recess size also impacts the number of turbinals that lie within the recess. Whereas only the posterior portions of ethmoturbinal II and III lie within the olfactory recess in *A. jamaicensis*, all ethmoturbinals and interturbinals are at least partially housed within the olfactory recess in the brachyphyllinis.

**Table 2:** Comparison of turbinal terminology used in this study, Bhatnagar and Kallen (1974, 1975) and Kämpfer and Schmidt (1977).

This study	Bhatnagar and Kallen (1974, 1975)	Kämpfer and Schmidt (1977)
Frontoturbinal	Ectoturbinal	Ectoturbinal I
Ethmoturbinal I	Endoturbinal I	Endoturbinal I
Interturbinal I	–	–
Ethmoturbinal II	Endoturbinal III	Endoturbinal II
Interturbinal II	Endoturbinal II	Ectoturbinal II
Ethmoturbinal III	Endoturbinal IV	Endoturbinal III

**Table 3:** Surface area and volume measurements of turbinals, as well as measurements scaled by cranial width.

		Length (mm)	Surface area (mm^2^)	Volume (mm^3^)	Surface area/skull	Volume/skull
*Artibeus jamaicensis*	Mastoid breadth	14.2				
	FT	3.4	37.5	3.2	1.22	0.10
	ET I	5.9	43.5	4.1	1.41	0.13
	IT II	2.7	40.8	3.0	1.33	0.10
	ET II	2.7	26.4	2.6	0.86	0.08
	ET III	2.8	32.5	2.0	1.05	0.07
	Total		180.7	14.9	5.87	0.48
*Erophylla bombifrons*	Mastoid breadth	11.0				
	FT	3.5	11.1	0.7	0.46	0.03
	ET I	5.4	23.1	1.5	0.96	0.06
	IT II	2.6	7.2	0.6	0.30	0.02
	ET II	3.5	11.9	0.8	0.49	0.03
	ET III	3.2	10.5	0.6	0.43	0.03
	Total		63.8	4.2	2.65	0.18
*Phyllonycteris poeyi*	Mastoid breadth	11.6^∗^				
	FT	4.5	22.8	2.0	0.92	0.08
	ET I	5.2	26.9	3.5	1.08	0.14
	IT II	2.8	7.1	0.7	0.29	0.03
	ET II	4.4	19.5	2.5	0.79	0.10
	ET III	5.4	27.2	2.1	1.09	0.08
	Total		103.6	10.8	4.16	0.43
*Brachyphylla pumila*	Mastoid breadth	13.0				
	FT	4.9	30.1	3.6	1.02	0.12
	IT I	4.3	29.2	2.9	0.99	0.10
	ET I	5.7	78.0	9.0	2.65	0.31
	IT II	4.1	24.9	2.3	0.84	0.08
	ET II	4.7	27.9	2.8	0.95	0.09
	ET III	4.4	19.8	1.7	0.67	0.06
	Total		210.0	22.2	7.12	0.75

*^∗^The head of Phyllonycteris poeyi was not completely scanned, and we thus could not accurately measure mastoid breadth. We therefore took the mean value reported in a previously published analysis (Mancina, 2010). Note that surface area based on surface models is not comparable to surface areas calculated in previous histological studies (Bhatnagar and Kallen, 1974, 1975; Eiting et al., 2014). Cranial width refers to greatest mastoid width, nasal cavity length measured between cribriform plate and naris. ET I, ethmoturbinal I; ET II, ethmoturbinal II; ET III, ethmoturbinal III; FT, frontoturbinal; IT I, interturbinal I; IT II, interturbinal II.*

**Table 4:** Length measurements of transverse lamina, nasal cavity, and skull.

	*Artibeus jamaicensis*	*Erophylla bombifrons*	*Phyllonycteris poeyi*	*Brachyphylla pumila*
Transverse lamina (mm)	2.4	2.9	4.6	4.3
Nasal cavity length (mm)	14.3	12.1	14.6	14.7
Greatest skull length (mm)	30.8	24.1	24.9^∗^	29.5
*Ratio to nasal cavity:*				
Transverse lamina	0.17	0.24	0.32	0.29
FT	0.24	0.29	0.31	0.33
IT I	–	–	–	0.29
ET I	0.41	0.45	0.37	0.39
IT II	0.19	0.21	0.19	0.28
ET II	0.19	0.24	0.30	0.32
ET III	0.20	0.26	0.37	0.30

*^∗^The head of Phyllonycteris poeyi was not completely scanned, and we thus could not accurately measure greatest length of skull. We therefore took the mean value of greatest skull length reported in a previously published analysis (Yohe et al., 2015). Turbinal proportions are calculated from values in **Table 3**. ET I, ethmoturbinal I; ET II, ethmoturbinal II; ET III, ethmoturbinal III; FT, frontoturbinal; IT I, interturbinal I; IT II, interturbinal II.*

Within Brachyphyllini, *B. pumila* differs from *E. bombifrons* and *P. poeyi* in the number and complexity of olfactory turbinals. *B. pumila* has two interturbinals (instead of one), the ethmoturbinal I is more elaborate and has two epiturbinals (additional turbinals that are attached to the ethmoturbinal), and the olfactory turbinals are more densely packed in the nasal cavity. *B. pumila* has the largest olfactory turbinal surface area to skull width and olfactory turbinal volume to skull width ratios of the four species (see **Table 2**).

## Discussion

Connecting morphology to the molecular underpinnings of trait loss may be informative toward understanding evolutionary mechanisms of phenotype loss, but methodological limitations have limited the scale to which this connection can be made across species. In this study, we use pioneering staining methods to bridge morphological with genetic evidence for the loss of function of the mammalian vomeronasal system in bats. Although histological and genetic data show that nearly all New World leaf-nosed bats (phyllostomids) have a well-developed vomeronasal system, variation in function within this family has emerged with recent molecular data (Yohe et al., 2017). We tested if a small clade of Caribbean nectar-feeding bats that shared an ancestral loss-of-function mutation in the vomeronasal signal transduction gene TRPC2 also lacked vomeronasal morphology. We established that both the vomeronasal organ and the epithelium lining the nasal cavity are detectable in a phyllostomid known to have an intact vomeronasal organ and intact TRPC2 using a non-invasive diceCT approach. Caribbean nectar-feeding bats lacking an intact TRPC2 have highly degraded vomeronasal morphology despite the apparent use of pheromone-mediated behaviors. Our data indicate that these brachyphyllines have well-developed olfactory recesses that are amongst the largest in relative length of all phyllostomids, especially compared to closely-related nectarivorous bats. This distinction opens up new interpretation of alternative pheromone-processing anatomical adaptations in light of vomeronasal loss.

Morphological studies of the mammalian chemosensory system have historically been limited to histology (Cooper and Bhatnagar, 1976; Bhatnagar and Meisami, 1998; Farbman, 2000). These studies require destruction of the specimen, which may hinder analyses of rarely sampled lineages. DiceCT has recently been shown to serve as a non-invasive technique to study soft-tissue morphology (Gignac and Kley, 2014; Gignac et al., 2016). Based on our diceCT scans, we detected and reconstructed the vomeronasal organ, including the vomeronasal epithelium and lumen, which was previously only thought to be possible through destructive serial sectioning. The measurement of vomeronasal length for *A. jamaicensis* is comparable to that of the length obtained from histology (Cooper and Bhatnagar, 1976). In short, diceCT is a sufficient method to non-invasively diagnose the presence or absence of vomeronasal anatomy, and to obtain continuous measurements. We also detected differences in the epithelial lining of the nasal cavity, possibly indicative of olfactory and respiratory epithelium. However, we only had histological sections of *A. jamaicensis* from a different individual and as such, we could not reliably test whether differences in epithelial lining seen in the μCT scans of brachyphyllines correspond to the two tissue types (Eiting et al., 2014). A more in depth histological comparison with complementary diceCT scans for several species will be advantageous to further demonstrate diceCT as a non-destructive approach for quantifying olfactory epithelium. We do not suggest diceCT as a replacement for invaluable histological sectioning, rather diceCT can be used to identify the presence or absence of the vomeronasal organ on a macroscopic level. The fine anatomical detail (e.g., neuronal morphology, presence of cilia, basal vs. apical epithelia, etc.) necessary to confirm functionality of a structure can, at least for now, only be obtained through microscopic histological sections. Furthermore, detection of the vomeronasal organ with diceCT does not indicate functionality with complete certainty, as vestigial structures can still be detectable. Nevertheless, diceCT enables the study of rare species that cannot be destructively sampled and as a more feasible method for larger sample sizes. As a whole-mount imaging technique, diceCT allows for three-dimensional visualization of the intact organ.

TRPC2 has been used as a proxy for vomeronasal function for over a decade, as it is seemingly an important signaling protein for vomeronasal transduction and is a pseudogene in several mammals with vestigial vomeronasal systems (Zhang and Webb, 2003; Yu et al., 2010; Zhao et al., 2011; Yohe et al., 2017). Once we confirmed the vomeronasal organ was indeed visible in the diceCT scans, we could test whether TRPC2 function or loss matched vomeronasal morphology in phyllostomids. As expected, *A. jamaicensis* had a well-developed vomeronasal organ and an intact, highly conserved TRPC2 (**Figures 2A**, **3A**). *A. jamaicensis* and its relatives within the subfamily Stenodermatinae are well-known for having intact vomeronasal morphology (Wible and Bhatnagar, 1996), and previous studies have shown TRPC2 has been under strong purifying selection in this group since early bat diversification (Yohe et al., 2017). Our diceCT data show that brachyphyllines lack a well-developed vomeronasal organ, and that it is likely vestigial in these species. Interestingly, degeneration of the vomeronasal organ occurs prior to genetic loss of function, as the basal brachyphylline *B. pumila* has an intact TRPC2, but a rudimentary vomeronasal system comparable to *E. bombifrons* and *P. poeyi*. While it may be that the *Brachyphylla* TRPC2 contains a pseudogenizing mutation elsewhere in the gene (as only exon 2 is sequenced), the shared disruptive deletion occurred subsequently to divergence of *E. bombifrons* and *P. poeyi* from *B. pumila*. A recent study detected a synchronous negative relationship between codon substitutions in TRPC2 and accessory olfactory bulb presence (Yohe and Dávalos, 2018), and indeed predicts the absence of the vomeronasal brain region in brachyphyllines based on the high rates of protein-changing substitutions. However, this study helps to clarify that the complete loss of function of TRPC2 in Caribbean nectar-feeders is slightly decoupled from the loss of well-developed vomeronasal morphology. This suggests that anatomical vestigialization may have occurred prior to the pseudogenization of essential molecular mechanisms of the system.

Because the mainland phyllostomids are under strong purifying selection to maintain a functional vomeronasal system (Yohe et al., 2017), it is curious why selection would relax in insular brachyphyllines. This is especially puzzling given that brachyphyllines make use of chemically-mediated social behaviors during courtship and mating rituals, behaviors normally contributed to mediation by the vomeronasal system. For example, males in the genus *Erophylla* perform wing-flapping dances and spray garlic-scented secretions from the supraorbital glands when courting a female (Murray and Fleming, 2008), suggesting that social chemical cues are still being processed in light of absent vomeronasal anatomy and disrupted TRPC2. One hypothesis is that the main olfactory system may compensate for vomeronasal loss, as recent evidence suggests the main olfactory system is also capable of processing pheromonal cues (Hohenbrink et al., 2014). For example, in rodents, vomeronasal receptors are expressed in the main olfactory epithelium and relay signals to the main and accessory olfactory bulb (Keller et al., 2009; Suárez et al., 2012; Omura and Mombaerts, 2014). Vomeronasal receptors in non-phyllostomids have been described for two species that have a non-functional TRPC2 and they are all pseudogenes (Young et al., 2010), but the functionality of these receptors in phyllostomids, and particularly in brachyphyllines, is still not known. Some emballonurid bats, which lack a vomeronasal system, still detect mating chemical cues via major histocompatibility complex (MHC) and trace amine-associated receptor (TAAR) genes expressed in their main olfactory system (Santos et al., 2016). The main olfactory system indeed has several subsystems that have been demonstrated to respond to pheromonal cues such as social food preferences, predation cues, conspecific identification, or territorial-inducing compounds. The processing of these cues are mediated by receptors and ion channels that are alternative to vomeronasal receptors and TRPC2 (e.g., formyl peptide receptors, TRPM5 or GC-D^+^-expressing olfactory sensory neurons) (Lin et al., 2007; Liberles et al., 2009; Munger et al., 2010). The relevance of these systems in bats may be a fruitful avenue of research, as this group demonstrates evolutionary loss of vomeronasal function as opposed to what is demonstrated in rodent knockout studies.

We considered the possibility of the main olfactory system compensating for vomeronasal loss within brachyphyllines. If this were the case, we might expect a relative increase in the surface area of olfactory epithelia in the nasal cavity. However, rather than finding that brachyphyllines have higher ratio of olfactory turbinal surface area vs. skull width compared to *A. jamaicensis*, we found that there is extensive variation in the clade. *B. pumila* not only shows absolutely and relatively greater olfactory turbinal surface area compared to that of *A. jamaicensis*, but also has an additional turbinal (interturbinal I) (**Table 2** and **Figure 3**), suggesting increased olfactory epithelial surface area. Secondary evolution of new turbinals suggesting subsequent augmentation of olfactory mucosa has been observed before in the distantly related Old-World lineage, *Megaderma lyra* (Smith et al., 2012). In contrast, *E. bombifrons* has less than half of the absolute and relative surface area of that observed in *A. jamaicensis*, while *P. poeyi* showed slightly lower absolute and relative olfactory turbinal surface area compared to *A. jamaicensis*, suggesting decreased olfactory surface area in these taxa. The range of turbinal surface areas in these closely related species suggest that a straightforward increase in olfactory turbinal size is not compensating for vomeronasal loss.

It should be noted that we measured turbinal volume and turbinal surface area, but not olfactory epithelial surface area. Part of the nasal septum and olfactory recess is also lined with olfactory epithelium and, as such, turbinal size likely underestimates the true extent of this tissue. This is particularly relevant for the brachyphyllines, as they share a relatively larger olfactory recess compared to *A. jamaicensis*. The olfactory recess is a cul-de-sac within the posterodorsal region of the nasal cavity that is defined ventrally by the transverse lamina. During sniffing, inspired air is directed to the olfactory recess where airflow is slowed, which may enable a more efficient delivery of odorant molecules to the chemoreceptors in the epithelial tissue (Eiting et al., 2014, 2015). In the brachyphyllines, the transverse lamina extends 24–31% of nasal cavity length, some of the largest proportions compared to other members of Phyllostomidae (Eiting et al., 2014). Further, the olfactory recess in these species contains large parts of the ethmoturbinals and interturbinals. In contrast, only the posterior-most aspect of ethmoturbinal II and III are positioned within the olfactory recess in *A. jamaicensis* (**Figure 3A**). Brachyphyllines drastically increase absolute surface area of olfactory turbinals, and potentially olfactory epithelium, in the olfactory recess by increasing the length of the recess and concentrating turbinals within the recess. Though the olfactory recess may play a role in slowing airflow and increasing deposition of odorants, it is unclear how this relates to olfactory ability. Eiting et al. (2015) found no correlation between the size of the olfactory recess and either olfactory bulb size or number of glomeruli (both commonly used as soft-tissue proxies for olfactory ability).

The presence of a large olfactory recess in Brachyphyllines is distinct from that of other nectar-feeding phyllostomids. Both *Anoura geoffroyi* and *Glossophaga soricina* exhibit among the smallest olfactory recesses within phyllostomids (Eiting et al., 2014). Brachyphyllines, as well the other nectarivorous phyllostomids have elongated palates (and a relatively long nasal cavity) that enables the consumption of nectar from the corollas of flowers. While the olfactory recess seems to decrease with palatal length in *A. geoffroyi* and *G. soricina*, the opposite is true for brachyphyllines. Interestingly, *A. geoffroyi* and *G. soricina* have functional and well-developed vomeronasal organs and intact TRPC2 (Yohe et al., 2017). Could the absence of a functional vomeronasal organ have an effect on size of the olfactory recess and the degree to which turbinals are enclosed by the transverse lamina?

Aside from the size of the olfactory recess, we further noticed a common shape in turbinal morphology within brachyphyllines that differs dramatically from that of *A. jamaicensis*. The Caribbean nectar-feeding clade shows elongated single-scroll frontoturbinals and interturbinals, whereas these turbinals are double-scrolls in *A. jamaicensis*. Though *G. soricina* likewise exhibits single-scroll turbinals, the turbinals in brachyphyllines are distinct in length (Kämpfer and Schmidt, 1977; Eiting et al., 2015:1). In all brachyphyllines, the olfactory turbinals are particularly elongated; the ethmoturbinal II extends between 29–32% the total length of the nasal cavity (**Table 4**). In stark contrast, the ethomoturbinal II appears to extend approximately 17% of the nasal cavity length in *G. soricina* (measured based on Kämpfer and Schmidt, 1977:1B).

Elongated turbinals and an enlarged olfactory recess may enhance the detection of pheromone cues in light of an absent vomeronasal organ. A more in-depth investigation of the olfactory recess, palate length, and vomeronasal presence across multiple species will unveil if there are constraining relationships among these variables, and this may be made possible through the use of diceCT to quantify these structures. Molecular markers such as TRPC2 are useful proxies for phenotypic function and loss, but only the comparative morphology of such phenotypes can reveal a complete picture of the mechanisms of loss of function. Using brachyphyllines as a model system to test whether genetic function of and underlying molecular mechanism are linked to the presence or absence of the vomeronasal system, our results suggest that genetic function is closely, but not entirely linked to morphological degradation of the sensory system. We also discovered unique turbinal morphology and nasal cavity anatomy that may compensate for brachyphylline pheromone-detection despite complete loss of vomeronasal function.

## Ethics Statement

This study was carried out in accordance with the recommendations of Stony Brook University Institutional Animal Care and Use Committee (SBU IACUC). The protocol was approved by the SBU IACUC.

## Data Availability

Surface models of reconstructions are available for download in the MorphoSource repository (project number: P494; media numbers: M23387, M23389, M23390, M23391). The raw data supporting the conclusions, including μCT-scan image stacks, of this manuscript will be made available by the authors, without undue reservation, to any qualified researcher.

## Author Contributions

LY and SH designed the study and interpreted the results. LY prepared the specimens. AC scanned the specimens. SH digitally reconstructed the vomeronasal organ and olfactory turbinals. SH and AC described the morphology. All authors wrote the manuscript.

## Conflict of Interest Statement

The authors declare that the research was conducted in the absence of any commercial or financial relationships that could be construed as a potential conflict of interest.

## Footnotes

1 www.volumegraphics.com
