# Supplementary material for: Vomeronasal and Olfactory Structures in Bats Revealed by DiceCT Clarify Genetic Evidence of Function
Source: Front Neuroanat. 2018 May 8;12:32. doi: 10.3389/fnana.2018.00032 (PMC5953337; doi:10.3389/fnana.2018.00032)
Supplement: Supplementary file 1 [file Data_Sheet_1.PDF]

Supplemental Material

Vomeronasal and Olfactory Structures in Bats Revealed by diceCT Clarify Genetic Evidence of Function

Authors: Laurel R. Yohe[1*], Simone Hoffmann[2*], and Abigail Curtis[3]
Affiliations:
[1]Department of Ecology & Evolution, Stony Brook University, Stony Brook, NY 11794, USA
[2]Department of Anatomy, New York Institute of Technology College of Osteopathic Medicine, Northern Boulevard, Old Westbury, NY 11568, USA
[3]Department of Biology, University of Washington, Seattle, WA 98195, USA
Correspondence:
Dr. Laurel R. Yohe
laurel.yohe@stonybrook.edu
Dr. Simone Hoffmann
shoffm04@nyit.edu

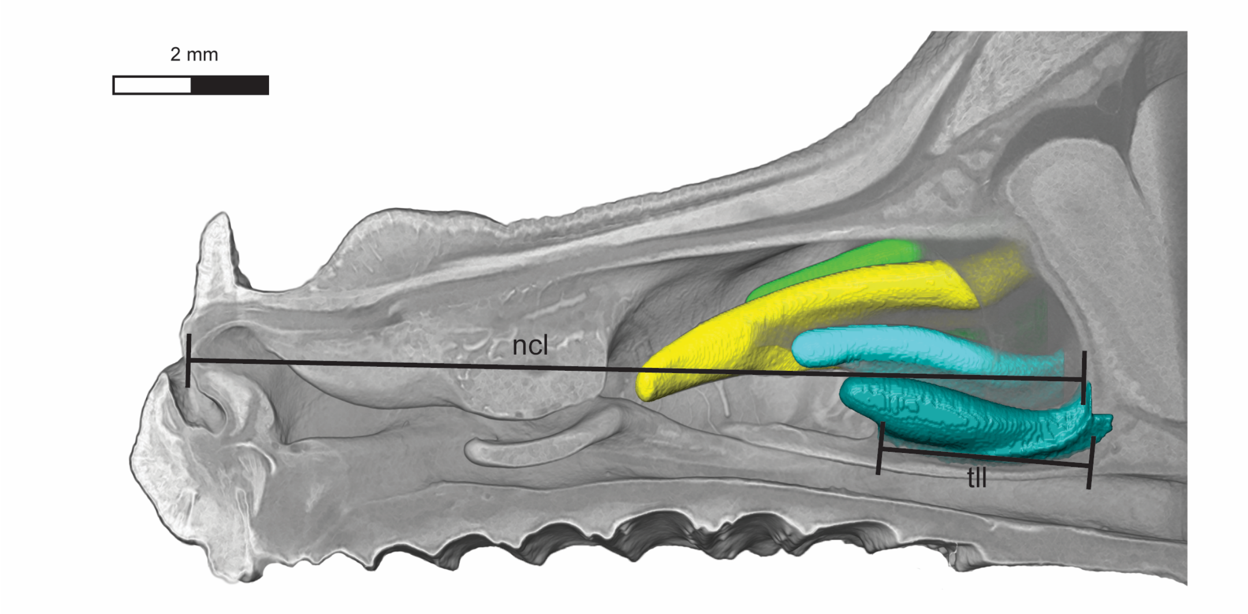

Figure S1. Lateral view (shown in *Erophylla bombifrons*) of scan to demonstrate linear measurements of the nasal cavity length (ncl) and transverse lamina length (tll).

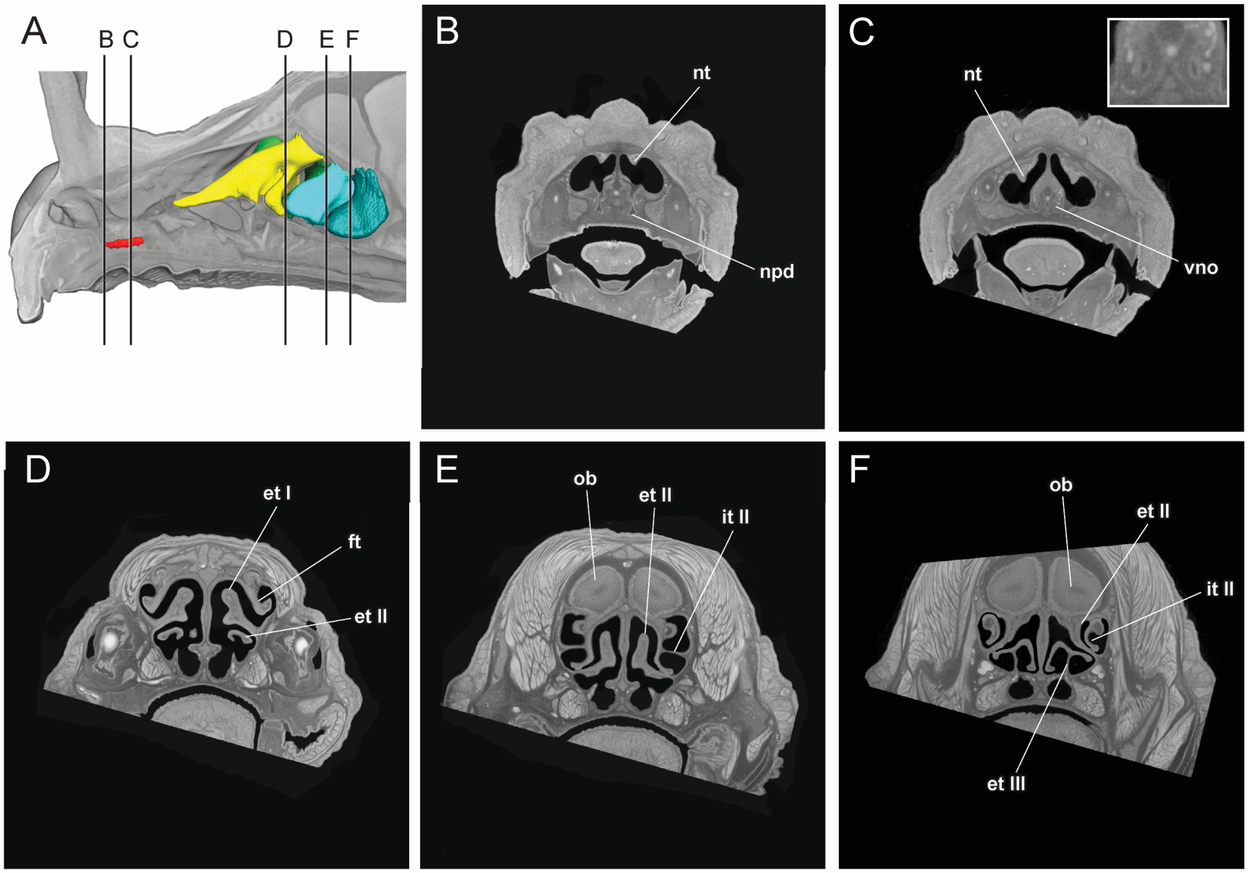

Figure S2. Serial coronal slices through the head of *Artibeus jamaicensis* obtained via μCT imaging. The position of each coronal slice (B–F) is indicated on the lateral side cut view of the head in A and refers to sections through (B) palatal opening of the nasopalatine duct, (C) posterior to the nasopalatine duct, (D) anterior end of the ethmoturbinal II, (E) anterior end of ethmoturbinal III, and (F) beginning of the olfactory recess. Abbreviations: ob, olfactory bulb; et I, ethmoturbinal I; et II, ethmoturbinal II; et III, ethmoturbinal III; ft, frontoturbinal; it II, interturbinal II; npd, nasopalatine duct; nt, nasoturbinal; vno, vomeronasal organ.

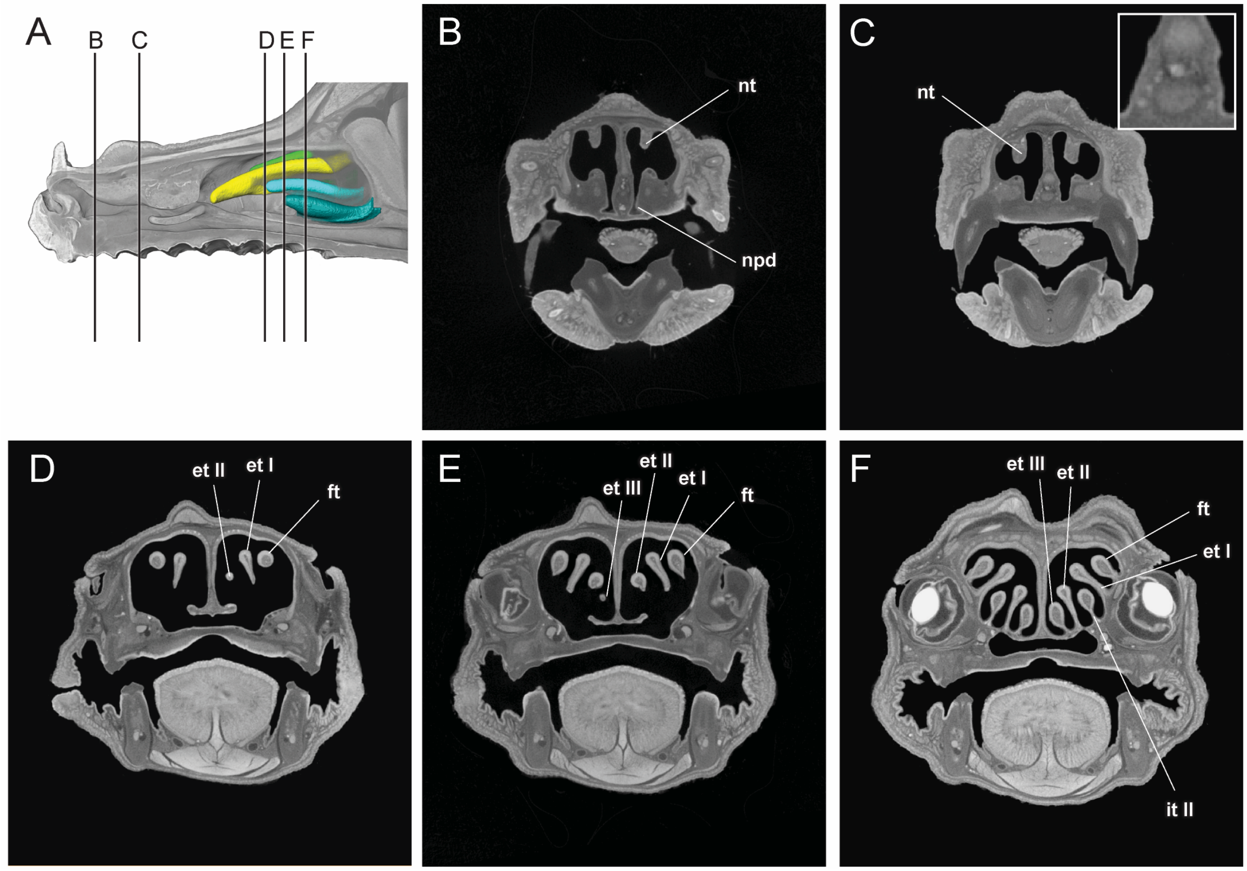

Figure S3. Serial coronal slices through the head of *Erophylla bombifrons* obtained via µCT imaging. The position of each coronal slice (B–F) is indicated on the lateral side cut view of the head in A and refers to sections through (B) palatal opening of the nasopalatine duct, (C) posterior to the nasopalatine duct, (D) anterior end of the ethmoturbinal II, (E) anterior end of ethmoturbinal III, and (F) beginning of the olfactory recess. Abbreviations: et I, ethmoturbinal I; et II, ethmoturbinal II; et III, ethmoturbinal III; ft, frontoturbinal; it II, interturbinal II; npd, nasopalatine duct; nt, nasoturbinal.

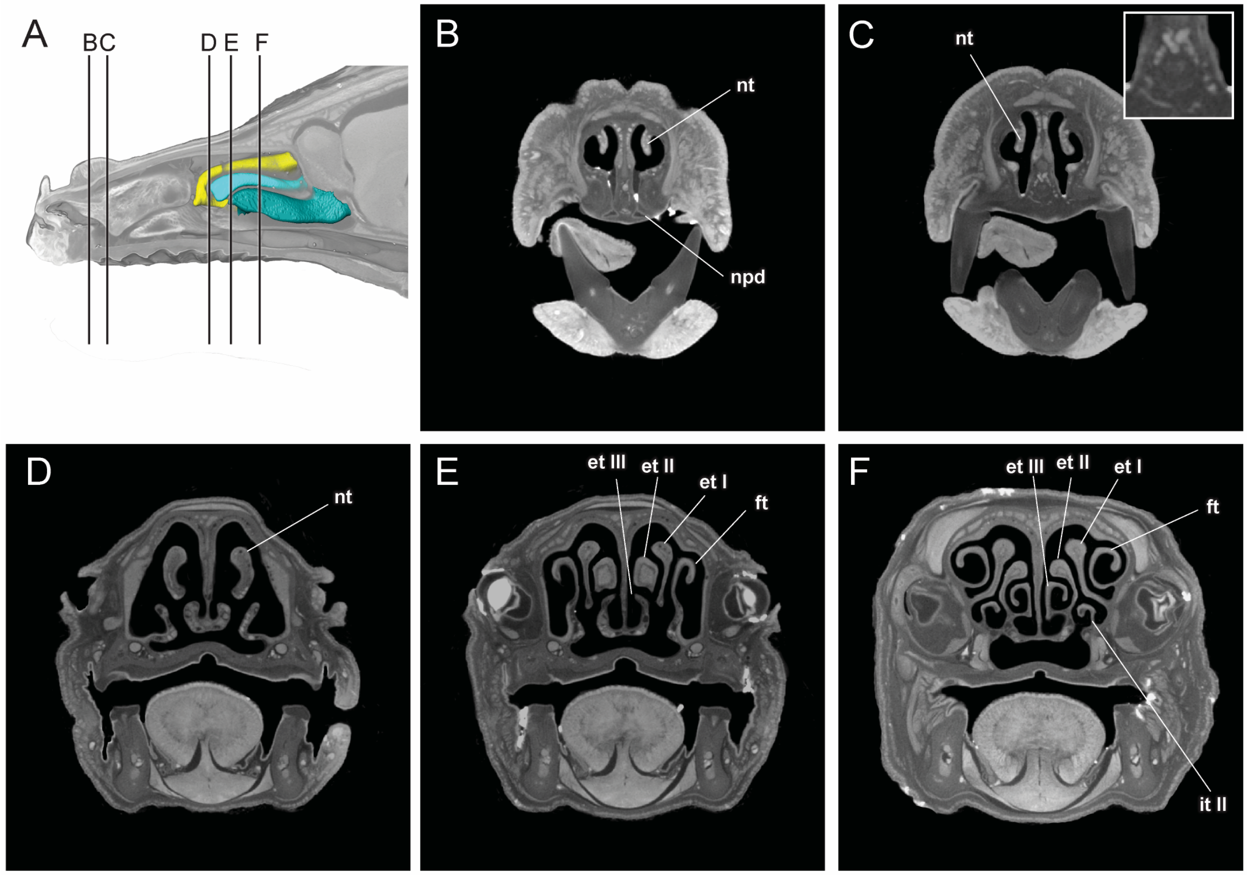

Figure S4. Serial coronal slices through the head of *Phyllonycteris poeyi* obtained via µCT imaging. The position of each coronal slice (B–F) is indicated on the lateral side cut view of the head in A and refers to sections through (B) palatal opening of the nasopalatine duct, (C) posterior to the nasopalatine duct, (D) anterior end of the ethmoturbinal II, (E) anterior end of ethmoturbinal III, and (F) beginning of the olfactory recess. Abbreviations: et I, ethmoturbinal I; et II, ethmoturbinal II; et III, ethmoturbinal III; ft, frontoturbinal; it II, interturbinal II; mt, maxilloturbinal; npd, nasopalatine duct; nt, nasoturbinal.

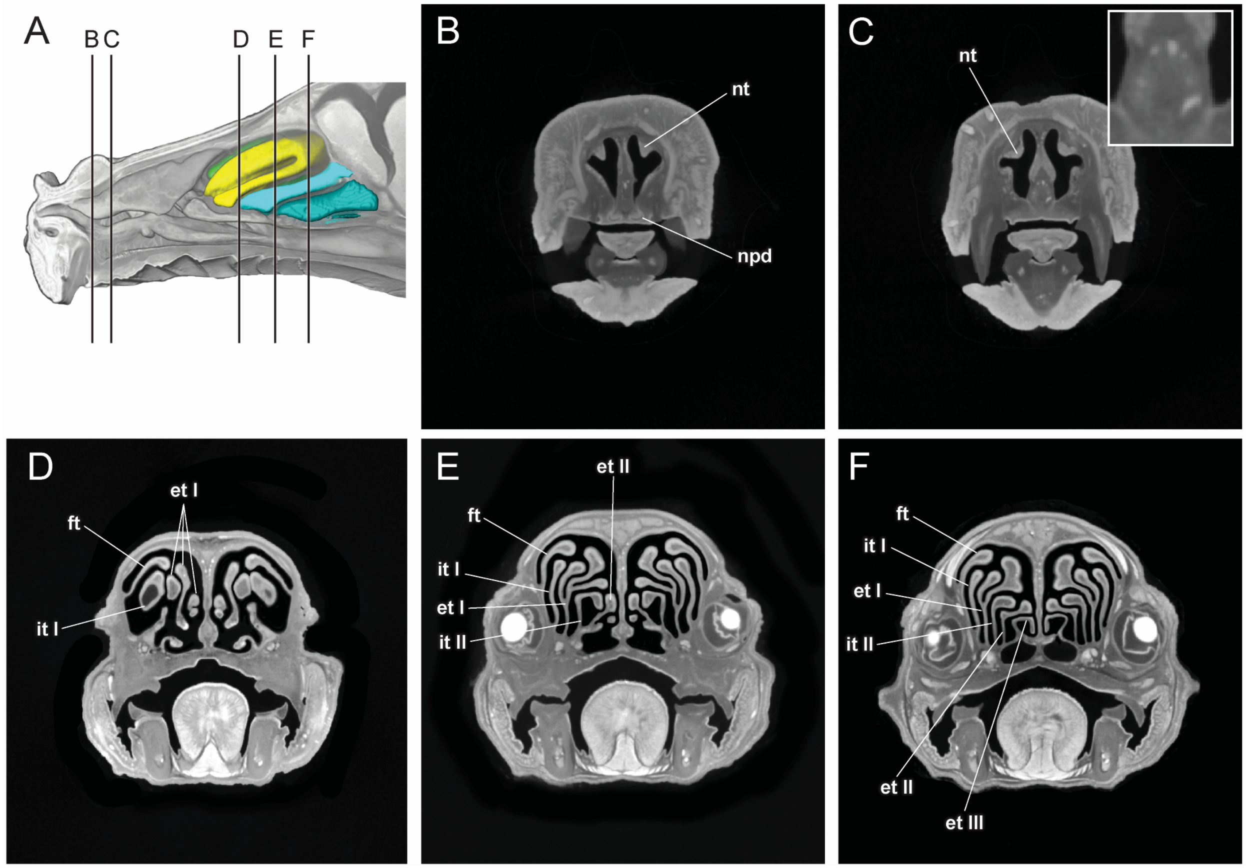

Figure S5. Serial coronal slices through the head of *Brachyphylla pumila* obtained via µCT imaging. The position of each coronal slice (B–F) is indicated on the lateral side cut view of the head in A and refers to sections through (B) palatal opening of the nasopalatine duct, (C) posterior to the nasopalatine duct, (D) anterior end of the ethmoturbinal II, (E) anterior end of ethmoturbinal III, and (F) beginning of the olfactory recess. Abbreviations: et I, ethmoturbinal I; et II, ethmoturbinal II; et III, ethmoturbinal III; ft, frontoturbinal; it I, interturbinal I; it II, interturbinal II; mt, maxilloturbinal; npd, nasopalatine duct; nt, nasoturbinal.
